# Cerebral Autoregulation and Neurovascular Coupling in Acute and Chronic Stroke

**DOI:** 10.3389/fneur.2021.720770

**Published:** 2021-09-03

**Authors:** Lucy C. Beishon, Jatinder S. Minhas

**Affiliations:** ^1^Department of Cardiovascular Sciences, University of Leicester, Leicester, United Kingdom; ^2^National Institute for Health Research (NIHR) Leicester Biomedical Research Centre, British Heart Foundation Cardiovascular Research Centre, Glenfield Hospital, Leicester, United Kingdom

**Keywords:** cerebral blood flow, dynamic cerebral autoregulation, cerebrovascular accident, cerebral haemodynamics, ischaemic stroke, intracerebral haemorrhage

## Introduction

Stroke is currently the second leading cause of death worldwide, and results in significant morbidity, and poorer quality of life for those affected (1). Stroke can be classified under two major sub-types: ischaemic and haemorrhagic. Ischaemic stroke accounts for ~70% of all stroke, and results from arterial occlusion, usually through embolism or small vessel thrombosis (2). Haemorrhagic stroke is a result of arterial rupture in the brain (2). However, the two sub-types frequently co-exist, with similar risk factors (e.g., hypertension), and overlap in pathological mechanisms (3).

Although stroke incidence has declined in high-income countries, it remains a prevalent issue amongst low and middle-income countries, disproportionately affecting a younger, working age population in these areas (1). The treatment of acute ischaemic stroke (AIS) has advanced over recent decades. Notably, the advent of both thrombolysis and mechanical thrombectomy has revolutionised the management of AIS, associated with reduced mortality, and improved functional outcome (2, 4, 5). Despite these advances, the management of haemorrhagic stroke has lagged behind, and treatment options are largely confined to reversal of anticoagulants and intensive blood pressure (BP) lowering (2). Conversely, in AIS, the target for BP management remains uncertain, and trials have largely shown equivalence (6, 7), or harm (8), associated with aggressive BP management strategies. To understand the mechanistic implications of BP lowering in AIS, studies have investigated the temporal changes in cerebral autoregulation (CA) following stroke (9). In healthy states, CA maintains a constant cerebral perfusion, despite fluctuations in systemic BP (10). However, the ability of the brain maintain CA may be compromised in the acute phase of stroke, increasing the vulnerability of the brain to hypoperfusion with intensive BP management strategies (11, 12). Conversely, surges in BP during this vulnerable phase may risk haemorrhagic transformation of the infarct, resulting in poorer outcomes (11, 12). Thus, understanding the temporal nature of CA in the acute phase of stroke could provide important mechanistic insights to guide BP management strategies in the clinical setting.

A related concept to CA is the physiological mechanism of neurovascular coupling (NVC). Under healthy conditions, neuronal activity is tightly coupled to cerebral blood flow (CBF), such that increases in neuronal activity will result in increases in CBF to ensure the metabolic demands of the brain are met. Intact NVC is integral to maintain optimal cognitive function, and thus may be an important physiological mechanism in the chronic or rehabilitation phase of stroke. The following sections consider the evidence to support a role for CA and NVC as important mechanistic factors in the acute and chronic phases of stroke, and the key clinical and research implications going forward.

## CA

Dynamic cerebral autoregulation (dCA) has now been carefully characterised at rest in AIS (13), acute intracerebral haemorrhage (ICH) (14) and chronic stroke (15) states. Furthermore, several studies have modelled the relationship between arterial CO_2_ (PaCO_2_), cerebral blood flow and dynamic cerebral autoregulation (16, 17). Hypercapnia causes vasodilation and deteriorates CA, with hypocapnia conversely causing vasoconstriction and an improvement in CA status (16, 17). Meta-analyses, albeit with significant heterogeneity, have demonstrated transfer function analysis parameter [phase and autoregulation index (ARI)] impairment in large and small artery AIS, lower phase in ICH and “rebounding phase” in chronic stroke (13). Unfortunately, limitations of existing transcranial Doppler based haemodynamic studies include low assessment frequency post stroke [particularly lacking data in ultra-acute (hours) and medium to longer term (weeks to months)] and clarification of dCA “cut-points” for impairment. Until very recently, there was a lack of dCA data peri- mechanical thrombectomy (MT), however, recent studies have shown worse dCA in the first 24 h associated with higher rates of haemorrhagic transformation and lower rates of recanalization (18). Specific learnings from this data suggest incomplete recanalisation of large-vessel occlusion, with impaired autoregulation status confer complication—raising the importance of adequate blood pressure control in this context (18). Whilst there are confounders to consider when assessing dCA pre-, during or post- MT including blood pressure (19, 20), end-tidal carbon dioxide level (21) and mode of anaesthesia (22)—their behaviour and interactions are yet to be determined. Higher end-tidal CO_2_ levels in those with incomplete recanalisation, especially beyond 72 h post large-vessel occlusion (LVO) is of significant interest (18). In ICH, the storey differs, with severe hypocapnia (low arterial CO_2_ levels) associated with poor prognosis (23). Furthermore, lowering BP during acute hypertensive states during ICH, in the setting of low arterial CO_2_ levels, leads to a greater risk of ischaemic lesions on MRI imaging (23). These differences in acute haemodynamics between stroke sub-types could be explained by nature of structural lesion (infarct vs. haematoma), existence of pre-existing chronic hypertension or differing responses to blood pressure lowering. Given personalised autoregulation-based BP targets are now possible in both a ward based stroke setting (24) and neurocritical care (25). Unfortunately, there still remains an inability to quantify the potential modulation of dCA by chronic hypertension before, during and immediately after acute stroke. The perceived “rightward shift” in the dCA curve is yet to be proven in acute (within 96 h) and sub-acute (7 to 14 days) contexts with ongoing hypertension or antihypertensive treatment being administered (26).

Recent advancements have further highlighted the need to recognise inter-subject variability (27) and responders vs. non-responders (28). There is evidence to suggest dCA impairment is greatest in regions with critically reduced perfusion (greatest volume of viable tissue), though dCA impairment can be present across the entire hemisphere to varying degrees (27). In ICH, through routinely obtained MRI scans in the first 7 days post-event, initial BP, nadir BP, and arterial CO_2_ were independent predictors of diffusion-restricted lesion incidence (23). Pooled individual patient data meta-analyses from the ATACH-2 and MISTIE III trials demonstrated in a heterogeneous cohort of patients with ICH, diffusion-weighted imaging (DWI) lesions were associated with 2.5-fold heightened risk of stroke among ICH survivors—with elevated risk persisting for AIS but not for recurrent ICH (29). In order to determine whether ischaemic lesions noted on DWI are preventable, or indeed governed by therapeutic variation in BP approaches (30)—mechanistic dCA studies at time of BP lowering, with continuous end-tidal CO_2_ measurement are needed, with MRI DWI assessment at 7 days. White matter ischaemic change may be attributed to by high blood pressure variability in addition to adverse adaptations of CA. In hypertensives without acute stroke disease, dCA (assessed using ARI) and CO_2_ reactivity were not related to white matter lesions—however, relationships with duration of hypertension and nocturnal BP dipping were shown (31). Ultimately, there exists a complex interdependent relationship between acute and chronic hypertensive states, dCA, and chronic cerebrovascular ischaemic injury. Crucially, we have evidence to support the hypothesis that carbon dioxide change in the acute setting post-stroke may modify risk, through interaction with BP lowering and dCA status, increasing the ischaemic stroke risk post ICH (23).

In both AIS and ICH, there exist adverse pathophysiologically driven complications including vasogenic oedema and haematoma expansion, respectively. The behaviour of cerebrovascular tone (critical closing pressure, CrCP) and resistance (resistance area product, RAP) is less well-understood. There is debate as to the sensitivity of CrCP to variation in intracranial pressure (ICP) (32). However, the presence of a haematoma in ICH as compared to controls, during normocapnic and hypocapnic conditions, showed significant differences in CrCP and RAP (33). Beyond common indices of dCA, there is limited knowledge of tone and resistance parameters in acute cerebrovascular states as compared to the traumatic brain injury literature.

## NVC

To date, the majority of studies have focussed on changes in CA in the acute, subacute, and chronic phases of stroke, with fewer studies investigating the effects on NVC (34). Animal models suggest that NVC is impaired early after stroke as a result of the reduction in neural activity which drives increases in CBF via feed forward mechanisms under normal conditions (35). In a mouse model of stroke, NVC processes were disrupted early after small-scale stroke, with disturbances peaking in the subacute period post-infarction, and remaining in the chronic phase (8 weeks post-event) (36). Impairments have been found to be widespread, occurring beyond the site of initial infarction (35, 36), and recovery of neural activity lags behind the restoration of perfusion (36). Perfusion in the acute phase was found to be predictive of neuronal outcome and recovery, in keeping with clinical studies discussed below (36).

In a systematic review, sixteen studies investigated changes in brain activation using transcranial Doppler or positron emission tomography based imaging (34). The review found mixed findings, with variable changes in response in both the affected and unaffected hemispheres (34). However, studies varied in the paradigm used to evoke CBF responses (sensorimotor, word finding, object recognition, word repetition and reading tasks), the phase of stroke studied, and the imaging modality used (34). Thus, it remains unclear to what extent these mixed findings are as a result of the heterogeneity in the methods used to assess NVC in stroke (34). Salinet et al. found NVC responses were reduced bilaterally to a passive motor paradigm within 48 h of stroke onset, and this correlated with stroke severity, and poorer functional outcome at 3 months (37). In a separate analysis, this was found to be as a result of myogenic, rather than metabolic impairment in NVC mechanisms (38). In a functional magnetic resonance imaging (MRI) study of chronic stroke patients, motor activity was associated with increases in CBF and cerebral blood volume (CBV) on arterial spin labelling, but with no discernible blood-oxygen level dependent response (39). However, CBF and CBV responses were attenuated when compared to healthy adults suggesting persistent abnormalities in NVC in chronic stroke, but these were dependent on the imaging modality used (39). The effects of thrombolysis on NVC processes are not fully understood, but may be as a result of effects on endothelial N-methyl-D-Aspartate receptor signalling (40, 41). However, function at the neurovascular unit has been suggested as one mechanism for the variability in inter-individual outcome with thrombolysis (42). In particular, patency of the microvasculature is essential to the recovery of neuronal function, and the level of injury in the unit determines the outcome with thrombolytic therapy (42).

To date, the majority of human studies have been cross-sectional, and longitudinal studies investigating the temporal evolution of NVC changes post-stroke are lacking. In particular, the role of NVC in the pathogenesis and outcome in haemorrhagic stroke has not been researched. Available evidence suggests NVC disruption in the early phases is predictive of functional outcome in ischaemic stroke (36, 37, 43), however cognitive outcomes have not been widely studied. The majority of human studies have focussed on sensori-motor rather than cognitive paradigms (34), which may be more relatable to recovery of motor rather than cognitive function. Importantly, up to one third of patients after stroke will experience long terms problems with memory and cognition, and severe stroke can bring forward the onset of dementia by up to 25 years (44). However, the relationship between cognitive function and NVC disruption remains under-researched. Thus, significant gaps remain in our understanding, particularly concerning how NVC process may be modulated to enhance functional and cognitive recovery in patients after stroke.

## NVC and CA Recommendations

Given the evolution of the field and the desire to utilise haemodynamic studies to deduce treatment response, prognostic indices and optimise physiological profiles—there is an ever-increasing individualised approach. However, gaps have emerged across both NVC and CA lines of investigation, offering an opportunity to highlight necessary short- and medium-term study recommendations:

- What is the longitudinal behaviour of NVC beyond sub-acute stroke and into chronic stroke states?
- Does NVC behaviour differ post ICH as compared to AIS?
- Can NVC be modulated to enhance functional and cognitive recovery in patients post stroke?
- Is there a longitudinal relationship between CO_2_ change post stroke and development of white matter lesions?
- To what extent does BP lowering cause harm due to its interaction with hypocapnia in AIS and ICH?

In order to address these pending research questions, multi-modality and inter-disciplinary studies are necessary. In addition, pooling of existing datasets to minimise research data waste and to maximise validity and statistical power is essential. There already exist multi-centre efforts to this effect, across cerebrovascular (45) and non-cerebrovascular (46) disease states. We encourage those working within the field to support these initiatives.

## Author Contributions

LB and JM prepared and wrote this manuscript through equal contribution to all aspects of its delivery.

## Author Disclaimer

The views expressed in this publication are those of the author(s) and not necessarily those of the NHS, the National Institute for Health Research, or the Department of Health, or the authors' respective organisations.

## Conflict of Interest

The authors declare that the research was conducted in the absence of any commercial or financial relationships that could be construed as a potential conflict of interest.

## Publisher's Note

All claims expressed in this article are solely those of the authors and do not necessarily represent those of their affiliated organizations, or those of the publisher, the editors and the reviewers. Any product that may be evaluated in this article, or claim that may be made by its manufacturer, is not guaranteed or endorsed by the publisher.
